# Rethinking Restrictive Diets in IBD: Toward an Inclusion-Focused Nutritional Paradigm

**DOI:** 10.1007/s11894-026-01047-0

**Published:** 2026-09-19

**Authors:** Brandon Cunha, Ari Alter, Madeline Berschback, Sunhee Park, Stephanie Gold, Yecheskel Schneider

**Affiliations:** 1 Rowan-Virtua School of Osteopathic Medicine, Stratford, NJ USA; 2 Department of Internal Medicine, Virtua Health, Camden, NJ USA; 3 New York Medical College, Touro University, Valhalla, NY USA; 4 Division of Gastroenterology, Massachusetts General Hospital, Boston, MA USA; 5 Division of Gastroenterology, University of California – Irvine School of Medicine, Orange, CA USA; 6 Division of Gastroenterology, Mount Sinai Hospital, New York, NY USA; 7 Division of Gastroenterology, Virtua Health, Moorestown, NJ USA

**Keywords:** Inflammatory bowel disease, Elimination diet, Mediterranean diet, Nutritional therapy, ARFID, Dietary restriction, Microbiome, Malnutrition, Dietitian, Crohn's disease, Ulcerative colitis

## Abstract

**Purpose of Review:**

Patient-initiated elimination diets are widely adopted by those with inflammatory bowel disease (IBD), yet the evidence supporting their long-term use remains limited and their risks are increasingly recognized. Some common elimination diets utilized by patients include the specific carbohydrate diet (SCD), low-FODMAP diet, and gluten-free and dairy-free diets. This narrative review critically examines the role of self-initiated restrictive dietary strategies in IBD management, evaluating the evidence for and against common elimination-based approaches, and contrasts these with emerging data supporting more inclusive dietary patterns.

**Recent Findings:**

Sustained elimination diets carry meaningful risks: malnutrition, micronutrient deficiency, sarcopenia, gut microbial dysbiosis, food anxiety, and avoidant/restrictive food intake disorder (ARFID). Current evidence demonstrates that the Mediterranean diet performs comparably to more restrictive approaches in inducing symptomatic remission, and generally is not associated with adverse nutritional and psychosocial consequences. Contemporary recommendations from several gastrointestinal societies discourage long-term elimination diets and favor inclusive, nutritionally adequate patterns, particularly the Mediterranean diet, as a preferred dietary framework for most patients with IBD, though supporting evidence remains largely observational and randomized controlled trial data are limited.

**Summary:**

We propose a paradigm shift from dietary exclusion toward inclusion, diversity, and nutritional adequacy, with registered dietitians as essential partners in IBD care. When elimination is clinically indicated, it should be time-limited, goal-directed, and followed by structured food reintroduction. Moving beyond restriction represents not a retreat from dietary focus, but its maturation, aligning nutritional guidance with the principles of patient-centered, evidence-based disease management.

## Introduction

Ever since inflammatory bowel diseases (IBD) were first described, the role of diet in pathogenesis and treatment has been of strong interest. Prior to the availability of effective immunomodulatory and biologic therapies, dietary modification was frequently employed as a primary strategy to alleviate gastrointestinal symptoms. Early recommendations emphasized avoidance, including low-residue diets, fiber restriction, and elimination of foods believed to exacerbate inflammation or precipitate obstruction. These approaches were largely symptom-driven rather than disease-modifying, but they established a culture of dietary caution that persists to this day [1, 2]. 

Even with advancements in pharmacologic therapies, questions about what type of diet to follow remain. Eating is unavoidable, personal, and often a form of social interaction. Patients consistently identify diet as one of the few modifiable factors they feel direct control over, with surveys demonstrating that many patients modify their diets after diagnosis and impose food restrictions specifically to prevent relapses [3, 4]. This patient-centered focus on diet has intensified in recent years, reshaping expectations of nutritional therapy in IBD [1]. 

Since the 1980s, with the publication of Breaking the Vicious Cycle by Elaine Gottschall [5], elimination diets have moved into mainstream patient discourse. Diets such as the specific carbohydrate diet (SCD), low–fermentable oligo-, di-, and monosaccharides and polyols (FODMAP) diet, gluten-free and dairy-free diets, and various “anti-inflammatory” or “clean eating” regimens have become popular diets for those with IBD [1, 3, 6]. Many patients adopt these diets independently, often prior to formal dietary counseling or even before a definitive diagnosis is established [3, 4]. Several forces have driven this trend, including social media. Social media and online patient communities amplify anecdotal success stories and simplify complex disease processes into dietary cause-and-effect narratives [7, 8]. Wellness culture frequently frames restriction as inherently health-promoting, equating elimination with inflammation reduction [7, 8]. For patients wary of lifelong immunosuppression, dietary restriction may appear safer, more natural, and more empowering than taking medication [1, 3]. 

However, increasing adoption of elimination diets has been accompanied by growing concern among clinicians regarding the effectiveness, sustainability, and harmful consequences of long-term dietary restriction [1, 2, 9]. Multiple converging developments make reassessment of elimination diets in IBD both timely and necessary. First, randomized trial data challenge the assumption that greater dietary restriction confers clinical benefit. Head-to-head comparisons, most notably between the SCD and Mediterranean diet, have failed to demonstrate meaningful differences in remission rates or inflammatory biomarkers [10]. 

Second, accumulating evidence highlights the nutritional and psychological harms associated with prolonged restriction. Malnutrition, micronutrient deficiencies, sarcopenia, food anxiety, and avoidant/restrictive food intake disorder (ARFID) are increasingly recognized in patients adhering to highly restrictive diets [11–15]. In pediatric populations, where growth impairment is driven primarily by disease-related inflammation and malnutrition, unnecessary dietary restriction may pose additional and potentially compounding nutritional risk [13, 16, 17]. 

Third, advances in immunology have reframed our understanding of diet–immune interactions. Rather than focusing primarily on exclusion of perceived dietary “triggers,” contemporary evidence increasingly emphasizes dietary diversity, fermentable fiber intake, and short-chain fatty acid production as likely contributors to mucosal immune regulation, though causality in human disease remains incompletely established [18, 19]. Sustained restriction may paradoxically undermine these protective mechanisms, as reduced dietary diversity depletes the fermentable fiber intake necessary to sustain butyrate-producing commensals and short-chain fatty acid production [18–20]. 

Considering the above, expert recommendations have evolved to reflect these insights, increasingly favoring inclusive dietary patterns, most notably the Mediterranean diet, over prolonged elimination as the preferred long-term dietary framework for most patients with IBD [1, 9]. 

In this review, we examine the role of elimination diets in IBD management in light of evolving evidence and expert consensus. We will review the clinical data supporting and refuting common elimination diets, and explore the nutritional and psychosocial risks of sustained restriction. We contrast these findings with evidence supporting more inclusive dietary approaches and propose a patient-centered framework that emphasizes dietary liberalization, personalization, and multidisciplinary care. This review focuses primarily on patient-initiated and commonly adopted elimination diets rather than clinician-directed nutritional therapies such as exclusive enteral nutrition (EEN) or the Crohn’s Disease Exclusion Diet (CDED), which represent distinct evidence-based interventions with established roles in select clinical contexts.

### Current Guideline Positions and Emerging Consensus

The American Gastroenterological Association (AGA) has addressed dietary patterns in inflammatory bowel disease through a 2024 Clinical Practice Update and expert review, acknowledging both strong patient interest in diet-based interventions and the limited quality of available evidence [1]. The AGA emphasizes that dietary counseling should focus on promoting overall nutritional adequacy and symptom management rather than disease modification. Within this framework, the AGA encourages consideration of following a Mediterranean diet for most patients with IBD, unless there is a contraindication to doing so. In addition to well-established long-term cardiometabolic health benefits, adherence to a Mediterranean dietary pattern has been associated with favorable clinical outcomes, including improved quality of life and lower rates of active disease, and may support gut microbial diversity in patients with both UC and CD [1]. 

Current AGA guidance does not support long-term restrictive diets, such as the low-FODMAP diet, representing a shift from prior AGA guidance that had endorsed low-FODMAP diets for functional gastrointestinal symptoms in IBD [1, 21]. While a short-term low-residue and low-fiber diet may be reasonable for patients during symptomatic disease flares, the AGA recommends transition back to a Mediterranean dietary pattern for long-term management [1]. The low-FODMAP diet has been associated with reductions in beneficial gut organisms and disrupts key nutrients involved in gut epithelial health [20, 22]. The AGA notes that a diet low in red and processed meat may reduce ulcerative colitis flares, but has not been found to reduce relapse in Crohn’s disease [1]. 

The European Crohn’s and Colitis Organisation (ECCO), the International Organization for the Study of Inflammatory Bowel Disease (IOIBD), and the American College of Gastroenterology (ACG), have similarly addressed the role of diet in inflammatory bowel disease, consistently emphasizing dietary interventions as adjunctive rather than primary therapy. Across these societies, there is broad agreement that while dietary modification may improve symptoms, current evidence does not support elimination diets as a durable strategy for inducing or maintaining disease remission. Dietary strategies are framed primarily as supportive measures aimed at improving overall health rather than as substitutes for established medical treatment [9, 23–25]. 

The 2025 ECCO consensus on dietary management of IBD underscores the importance of maintaining adequate nutrition and avoiding unnecessary dietary restriction [9]. While short-term dietary modification may be appropriate in select clinical contexts, such as during active disease or in the presence of obstructive symptoms, ECCO notes that restrictive diets have not demonstrated sustained benefits for disease control and may increase the risk of malnutrition. Accordingly, ECCO emphasizes early involvement of a trained dietitian to individualize dietary counseling, ensure nutritional adequacy, and support long-term adherence to inclusive dietary patterns [9]. 

Similarly, IOIBD guidance highlights the limited and heterogeneous evidence supporting specific elimination diets in IBD [23]. IOIBD acknowledges that dietary interventions may play a role in symptom management or as adjunctive therapy in select patients but cautions against the use of restrictive diets as standalone treatment. The organization places particular emphasis on shared decision-making, careful monitoring for nutritional deficiencies, and the involvement of multidisciplinary care teams when dietary interventions are pursued [23]. 

ACG clinical guidelines for both Crohn’s disease and ulcerative colitis adopt a comparably conservative stance [24, 25]. While recognizing patient interest in diet-based strategies, ACG guidance does not recommend diet as primary therapy for active disease and advises against replacement of pharmacologic therapy with restrictive diets for disease control, particularly in moderate-to-severe IBD. Short-term restrictive dietary modification may be considered in select patients with mild disease or for symptom management but should be implemented with appropriate clinical oversight and nutritional support [24, 25]. 

Despite differences in emphasis and scope, recommendations from the AGA, ECCO, IOIBD, and ACG consistently prioritize dietary approaches that support overall health, minimize nutritional risk, and can be sustained long term, rather than highly restrictive regimens aimed at symptom control [1, 9, 23–25]. Within this evolving consensus, the Mediterranean diet has emerged as a potential acceptable diet for patients, given its favorable safety profile, broad health benefits, and relative ease of long-term adherence [1, 9, 26]. Importantly, this shift does not suggest that dietary interventions are ineffective or irrelevant in IBD care. Instead, it underscores a growing recognition that diet is best positioned as an adjunctive component of comprehensive disease management, implemented with flexibility, individualization, and professional dietary support [1, 9, 23].

## Evidence for Elimination Diets in IBD: Benefits and Limits

Elimination diets are frequently adopted by patients with inflammatory bowel disease (IBD) in pursuit of symptom relief, reduced inflammation, or a desire to avoid long-term medications [1, 3, 4]. Despite their popularity, the evidence supporting most elimination-based dietary strategies remains limited, heterogeneous, and focused primarily on short-term symptom-focused outcomes rather than objective markers of disease activity [1, 9, 23]. Available evidence and clinical considerations for the diets discussed in this article are summarized in Table 1.

**Table 1:** Summary of evidence and key risks for common elimination and therapeutic diets in inflammatory bowel disease

Diet	Highest-Quality Evidence	Symptom Outcomes	Objective Disease Markers	Key Risks	Refs
**Specific Carbohydrate Diet (SCD)**	DINE-CD RCT (Lewis et al., 2021; *n* = 191); N-of-1 trials (Kaplan et al., 2022; *n* = 54).	Symptomatic remission 46.5% at 6 weeks — not superior to Mediterranean diet (*p* = 0.77). No consistent improvement in symptoms or fecal calprotectin.	Fecal calprotectin response: 34.8%; CRP response: 5.4%. No evidence of mucosal healing. Calprotectin response similar to Mediterranean diet (*p* = 0.83).	Micronutrient deficiencies (vitamin D, calcium, thiamine, folate, iron). High withdrawal rates. Adherence declines significantly over time.	[10, 33]
Low-FODMAP Diet	RCTs: Cox et al., 2020 (*n* = 52); Bodini et al., 2019 (*n* = 55). Meta-analysis: Zhan et al., 2018; Ville et al., 2025 (5 RCTs, *n* = 224).	Adequate symptom relief: 52% vs. 16% control (*p* = 0.007). IBS severity score reduction: not statistically significant. QoL improved (IBDQ; Cox et al., 2020). Meta-analysis (Ville et al., 2025): reduced global IBS symptom severity (SMD − 0.56; 95% CI − 0.90 to − 0.23; *p* = 0.0009; low certainty evidence).	No effect on CD or UC disease activity scores. Fecal calprotectin: no significant difference vs. control (SMD − 0.20; *p* = 0.17). No evidence of mucosal healing.	Depletes Bifidobacterium spp. and F. prausnitzii. Reduces butyrate production. Psychosocial burden. Long-term microbiome dysbiosis risk.	[19– [22, 27, 35, 36]
Gluten-Free Diet (GFD)	No RCTs in IBD. Cross-sectional data only (Schreiner et al., 2019; *n* = 54 GFD patients of 1,254 total).	Self-reported symptom improvement in observational studies only. No objective outcome data available.	No data on disease activity, fecal calprotectin, CRP, or mucosal healing in IBD populations.	Lower intake of fiber, folate, B12, vitamin D, iron, calcium. Higher cost. Lower psychological well-being; higher anxiety and depression.	[2, 23, 37]
Dairy-Free Diet	2 small RCTs in UC maintenance (Cochrane review, Limketkai et al., 2019ᵃ; *n* = 77 total).	Clinical relapse at 12 months: 59% milk-free vs. 68% control (RR 0.83, 95% CI 0.60–1.15). No statistically significant difference.	Endoscopic and histologic relapse data insufficient for analysis. No evidence of objective disease improvement.	Risk of calcium and vitamin D deficiency.	[23, 38]
Mediterranean Diet	DINE-CD RCT (Lewis et al., 2021; *n* = 191). Prospective cohort: Chicco et al., 2021 (*n* = 142; 6-month intervention).	Symptomatic remission 43.5% at 6 weeks (equivalent to SCD, *p* = 0.77). Chicco: reduced active disease rates in UC (23.7% to 6.8%, *p* = 0.004) and CD (17.0% to 3.8%, *p* = 0.011) at 6 months.	Chicco: reduced CRP and fecal calprotectin at 6 months; reduced liver steatosis. DINE-CD: fecal calprotectin response 30.8%. No endoscopic/mucosal healing data from RCTs.	No significant nutritional risks. Improves BMI and body composition. Enhances microbiome diversity. Supports cardiovascular and metabolic health.	[9, 10, 26]

ᵃDairy-free diet: evidence is limited to observational data; the Cochrane review by Limketkai et al. found insufficient evidence to recommend routine dairy elimination in IBD [38]

^b^All diets should be implemented under the supervision of a registered dietitian to minimize the risk of nutritional deficiency, particularly in patients with active disease, prior resection, or pediatric IBD

*IBD* inflammatory bowel disease, *CD* Crohn’s disease, *UC* ulcerative colitis, *RCT* randomized controlled trial, *SCD* Specific Carbohydrate Diet, *FODMAP* fermentable oligosaccharides, disaccharides, monosaccharides, and polyols, *GF* gluten-free, *RR* relative risk, *SMD* standardised mean difference, *CI* confidence interval, *CRP* C-reactive protein, *IBDQ* Inflammatory Bowel Disease Questionnaire, *BMI* body mass index

### Specific Carbohydrate Diet

The specific carbohydrate diet (SCD) is among the most commonly used elimination diets in IBD [1]. It restricts complex carbohydrates, grains, most dairy products, and processed foods based on the hypothesis that poorly-absorbed carbohydrates promote dysbiosis and intestinal inflammation [1, 5]. Early evidence supporting the SCD consisted of small observational studies and pediatric case series reporting symptomatic improvement; however, these studies were limited by lack of control groups, short follow-up, and reliance on subjective outcomes [2, 9]. 

More rigorous evaluation was provided by the Diet to Induce Remission in Crohn’s Disease (DINE-CD) trial, a randomized controlled study comparing the SCD with a Mediterranean diet in adults with mild to moderate Crohn’s disease [10]. Over 12 weeks, there were no significant differences between dietary groups in rates of symptomatic remission or inflammatory biomarkers (including fecal calprotectin). Importantly, the Mediterranean diet, which is far less restrictive, performed equivalently while offering greater dietary diversity and feasibility. These findings challenge the premise that greater dietary restriction confers superior anti-inflammatory benefit. Adherence declined over time in both dietary groups in DINE-CD [1, 10]. 

### Low-FODMAP Diet

The low–fermentable oligo-, di-, and monosaccharides and polyols (FODMAP) diet was developed for irritable bowel syndrome and targets poorly absorbed carbohydrates that increase luminal fermentation and gas production [2]. In patients with IBD, randomized trials demonstrate that low-FODMAP diets improve overall symptom relief and health-related quality of life in those with quiescent disease and IBS-like symptom overlap, though improvements in individual symptom domains such as abdominal pain did not consistently reach statistical significance [22, 27]. Even with the symptomatic benefits that do occur, there are not consistent improvements in objective inflammatory markers, including fecal calprotectin and C-reactive protein [22, 27]. Additionally, evidence suggests worsening dysbiosis with sustained restriction. Randomized trial data have demonstrated that low-FODMAP restriction significantly depletes Bifidobacterium species and Faecalibacterium prausnitzii, generally considered beneficial organisms central to butyrate production, epithelial barrier integrity, and intestinal immune regulation [19, 20, 22]. Accordingly, contemporary guidance recommends low-FODMAP diets only as short-term, symptom-directed interventions, followed by structured reintroduction and transition to a more inclusive dietary pattern [1, 21]. 

### Gluten-Free, Dairy-Free, and Other Elimination Diets

Gluten-free and dairy-free diets are frequently adopted by patients with IBD, often in the absence of celiac disease, lactose intolerance, or objective evidence of food sensitivity [3, 4]. To date, there is no high-quality evidence demonstrating that gluten avoidance improves inflammatory outcomes in IBD in patients without celiac disease [2, 23]. Similarly, dairy avoidance may alleviate symptoms in patients with lactose intolerance but does not appear to influence disease activity for those with IBD, as noted in both AGA and IOIBD dietary guidance [1, 23]. Other popular elimination-based dietary patterns, including autoimmune protocol diets, lectin-free diets, and broadly defined “anti-inflammatory” diets, lack robust clinical trial data in IBD [1, 6, 23]. Their theoretical frameworks are often extrapolated from non-IBD populations, and the potential risks of unnecessary dietary restriction are significant [1, 11, 23]. 

### Summary

Across elimination diet studies, a consistent theme is the disconnect between symptom improvement and objective disease control [1, 10]. While dietary restriction may reduce luminal triggers of bloating or diarrhea, symptom improvement does not reliably translate into mucosal healing, sustained biomarker normalization, or a reduction in flares [1, 10, 22]. In IBD, where symptoms correlate imperfectly with the underlying inflammatory burden, reliance on symptom response alone may provide false reassurance and delay appropriate medical therapy [1, 2]. Overall, elimination diets may provide short-term symptomatic benefit for select patients but lack evidence supporting long-term disease modification [1, 9, 23]. These limitations underscore the importance of weighing potential benefits against nutritional, psychosocial, and mechanistic risks [1, 11, 20]. 

## Limitations, Harms, and Risks of Restrictive Diets in IBD

Although elimination diets are often pursued with the intention of improving disease control or minimizing medication exposure, growing evidence highlights meaningful risks associated with sustained dietary restriction in IBD [1, 9, 23]. These harms span nutritional, psychosocial, microbiome-related, and feasibility domains and are particularly concerning given the lack of consistent long-term benefit [1, 12, 13]. These challenges are compounded by food insecurity, which affects approximately 13% of IBD patients and further limits the feasibility of adhering to specialized dietary regimens [28, 29]. 

### Nutritional Risks and Malnutrition

Malnutrition is a prevalent and underrecognized complication of IBD, affecting patients across disease phenotypes and degrees of activity [13, 30]. Restrictive diets may exacerbate this risk by limiting caloric intake, reducing nutrient density, and excluding entire food groups without clear medical indication [23]. Patients adhering to elimination diets are at increased risk for micronutrient deficiencies, particularly iron, vitamin B12, folate, vitamin D, zinc, and calcium [1, 13, 30]. 

Importantly, malnutrition may occur even in the absence of overt weight loss [12]. Restrictive eating patterns can preserve body weight while promoting unfavorable changes in body composition, including loss of lean muscle mass and sarcopenia. Sarcopenia has been associated with increased hospitalization, postoperative complications, decreased response to biologic therapy, and reduced quality of life in IBD populations [12, 31]. Pediatric patients are particularly vulnerable. Children and adolescents with IBD have increased nutritional requirements to support growth and pubertal development, and prolonged dietary restriction may contribute to growth impairment, delayed puberty, and suboptimal bone mineralization [13, 14, 16, 17]. 

### Psychosocial and Behavioral Harms

Restrictive diets may also have substantial negative psychosocial and behavioral effects. Food anxiety, hypervigilance around eating, and rigid dietary rules are commonly reported among patients with IBD adhering to elimination diets [11, 15]. Of particular concern is the association between elimination diets and avoidant/restrictive food intake disorder (ARFID), where restrictive intake is driven by fear of aversive consequences (such as pain or diarrhea), sensory sensitives, or lack of interest in eating [14, 15]. The medicalization of eating, where foods are labeled as inherently “safe” or “dangerous,” may inadvertently legitimize disordered eating behaviors [11, 14]. Patients may resist food reintroduction even during remission, interpreting normal gastrointestinal variability as evidence of disease harm [14, 15]. Over time, this rigidity may decrease quality of life and contribute to social isolation [11, 15]. 

### Microbiome and Immune Consequences

Dietary diversity is a key determinant of gut microbial diversity and resilience [18, 20]. Restrictive diets, especially those low in fermentable fibers, are associated with reduced abundance of beneficial commensal bacteria and decreased production of short-chain fatty acids, including butyrate [18–20]. Butyrate is thought to play an important role in epithelial barrier integrity and immune regulation, though the precise mechanisms and extent of this relationship in IBD is not fully elucidated [19]. 

### Feasibility, Cost, and Sustainability

Elimination diets frequently impose substantial financial and logistical burdens [27, 32]. Specialized foods, supplements, and increased meal preparation time may limit accessibility and adherence, particularly among patients with food insecurity or limited resources [1, 30]. High dropout rates across dietary intervention trials underscore the sustainability challenges of elimination approaches. This is most strikingly illustrated by the PRODUCE trial, in which 44% of participants withdrew from the specific carbohydrate diet arm [33]. A 2026 meta-analysis of 62 IBD dietary trials confirmed an overall non-completion rate of approximately 16% [34]. The stark contrast in completion rates between trials that provided prepared meals (such as DINE-CD) and those requiring self-preparation (such as the PRODUCE trial) suggests that real-world adherence to elimination diets may be substantially lower than even trial data indicate [10, 33]. 

## Evidence Supporting Inclusive Dietary Patterns

In contrast to elimination-based approaches, emerging evidence supports inclusive diets that emphasize nutritional adequacy, dietary diversity, and long-term sustainability [1, 9, 23]. These approaches align more closely with contemporary understanding of diet–microbiome–immune interactions and address many of the limitations inherent to restrictive diets [1, 9, 18, 19, 23]. 

### Mediterranean Diet

**Fig. 1:**
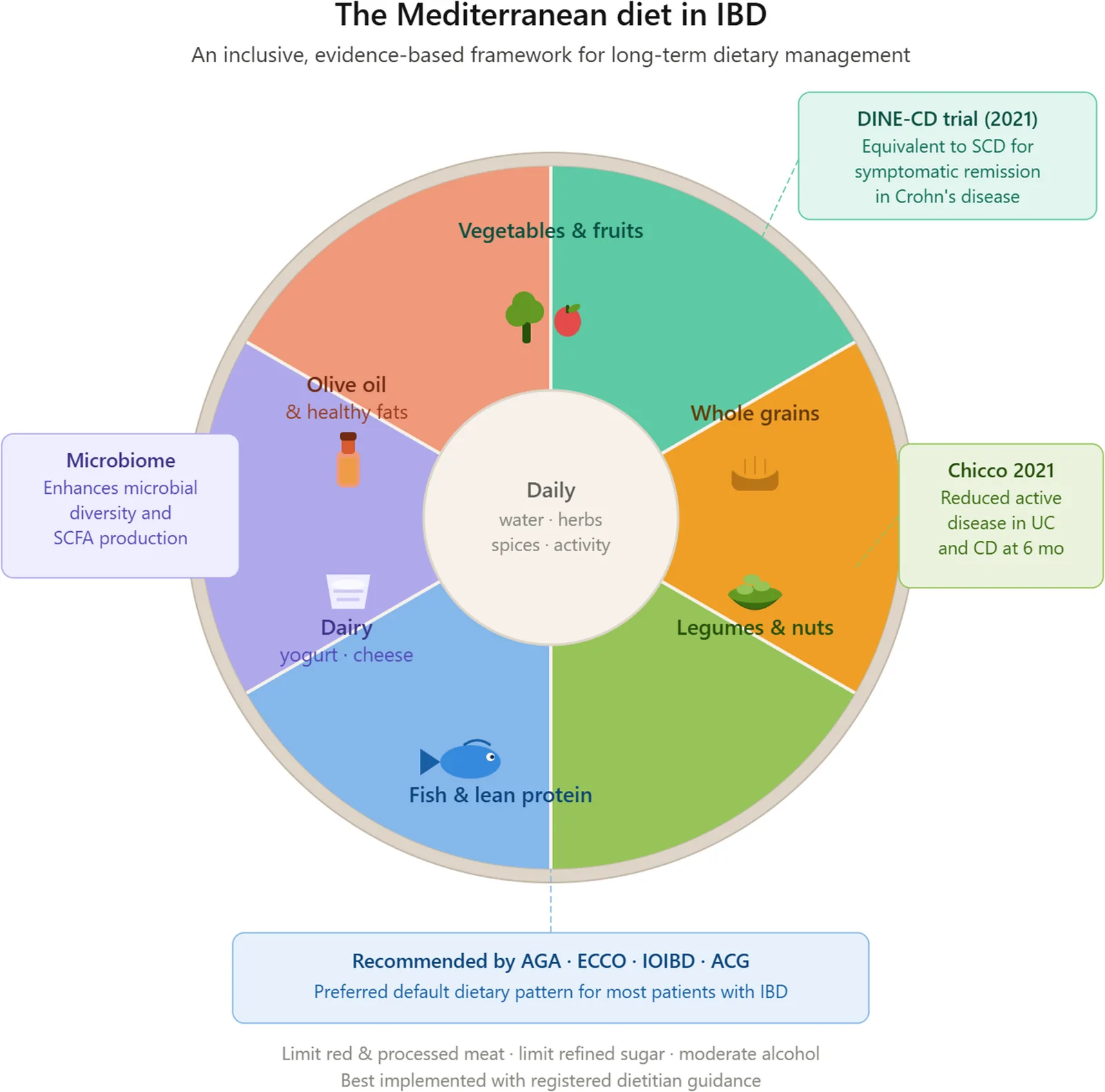
The mediterranean diet as a default dietary framework in inflammatory bowel disease. The Mediterranean diet emphasizes vegetables and fruits, whole grains, legumes and nuts, fish and lean protein, fermented dairy, and olive oil as primary fat source, with limitation of red and processed meat, refined sugars, and alcohol. Evidence supporting its use in IBD includes the DINE-CD randomized controlled trial, which demonstrated equivalence to the Specific Carbohydrate Diet for symptomatic remission in Crohn’s disease, and a prospective cohort study by Chicco et al. demonstrating reduced active disease in both Crohn’s disease and ulcerative colitis at six months. The Mediterranean diet is endorsed as a preferred dietary pattern by the American Gastroenterological Association, the European Crohn’s and Colitis Organization, the International Organization for the Study of Inflammatory Bowel Disease, and the American College of Gastroenterology. AGA, American Gastroenterological Association; ECCO, European Crohn’s and Colitis Organisation; IBD, inflammatory bowel disease; IOIBD, International Organization for the Study of Inflammatory Bowel Disease; ACG, American College of Gastroenterology; SCD, Specific Carbohydrate Diet; SCFA, short-chain fatty acid

Among inclusive dietary patterns studied for IBD, the Mediterranean diet is recommended by contemporary societies as a preferred dietary framework for most patients, though it is important to acknowledge that supporting evidence derives largely from observational studies, and randomized controlled trial data remain limited [1, 9, 26]. The Mediterranean diet is characterized by high intake of fruits, vegetables, whole grains, legumes, nuts, olive oil, and lean proteins (with an emphasis on fish), with limited consumption of red and processed meats. It is thought to support microbial diversity and anti-inflammatory metabolic pathways [1, 18, 26]. Some studies have demonstrated associations between Mediterranean diet adherence and improvements in disease activity indices, inflammatory biomarkers, and quality of life in both Crohn’s disease and ulcerative colitis [26]. Conversely, DINE-CD demonstrated that the Mediterranean diet did not improve markers of disease activity but did perform equivalently to the SCD for inducing symptomatic remission [10]. Beyond IBD-specific outcomes, the Mediterranean diet confers well-established cardiovascular and metabolic benefits, making it particularly well suited for long-term disease management [1, 26]. The core components of the Mediterranean diet and supporting evidence are illustrated in Fig. 1.

### Other Diets

Other dietary strategies, such as exclusive enteral nutrition (EEN), the Crohn’s Disease Exclusion Diet (CDED), and the IBD Anti-Inflammatory Diet (IBD-AID), represent clinician-directed, evidence-based therapeutic interventions that are fundamentally distinct from the patient-initiated elimination diets discussed throughout this review. EEN, in which all oral nutrition is replaced by a defined liquid formula, functions as first-line induction therapy for pediatric Crohn’s disease. EEN achieves mucosal healing rates comparable to corticosteroids while avoiding the growth-suppressive and immunosuppressive adverse effects of steroid therapy, making it particularly well-suited to pediatric and adolescent patients [39]. Its proposed mechanisms include alterations in luminal microbial composition, reduced antigenic stimulation, and restoration of intestinal barrier function, though definitive mechanistic pathways in humans remain under investigation. The CDED was developed in part as a more sustainable alternative to EEN. The CDED combines specific food inclusions and exclusions with partial enteral nutrition and has demonstrated efficacy for inducing remission in pediatric Crohn’s disease in randomized trial data, with studies showing benefit in adult disease as well [40]. The IBD-AID incorporates a phased dietary framework emphasizing prebiotic and probiotic foods, restriction of certain carbohydrates, and progressive dietary liberalization. This review does not intend to dismiss these structured dietary therapies, which are implemented under clinical supervision, have defined endpoints, and incorporate explicit reintroduction phases. When implemented under appropriate oversight, these interventions may offer meaningful benefit while maintaining a clear trajectory toward dietary liberalization [1, 9, 23]. 

### A New Paradigm: From Elimination to Inclusion in IBD Dietary Care

The cumulative limitations and harms of sustained dietary restriction, coupled with the absence of consistent disease benefit, necessitate a reframing of dietary care in IBD [1, 9, 23]. Rather than positioning exclusion as the primary tool for dietary therapy, emerging evidence supports a paradigm that emphasizes inclusion, diversity, and nutritional adequacy. Within this framework, elimination, when used at all, is best conceptualized as a temporary, targeted strategy embedded within a broader goal of liberalized, sustainable eating and medical therapy [1, 9, 23]. 

Elimination diets may retain a legitimate and meaningful role in select clinical scenarios, and the shift toward inclusion should not be interpreted as dismissing dietary restriction categorically. Specific contexts in which structured elimination or modification may be appropriate include: short-term symptom management in patients with IBD/IBS overlap, where low-FODMAP restriction can provide meaningful functional relief; diagnostic trials for suspected food intolerance in patients with persistent symptoms despite established disease control; temporary texture and fiber modification during active luminal inflammation, high-grade stricturing disease, or the perioperative period; and clinician-directed use of evidence-based dietary therapies such EEN or CDED when appropriate. In these contexts, elimination should remain time-limited, accompanied by explicit reintroduction planning, and implemented with dietitian involvement to minimize nutritional risk [2, 22]. Without safeguards, temporary restriction may become indefinite, increasing the risk of nutritional deficiencies [12, 13], food anxiety and disordered eating.

Central to this paradigm shift is the integration of registered dietitians into routine IBD care [1, 9, 23]. Dietitians provide expertise in nutritional assessment, micronutrient monitoring, and individualized counseling that cannot be replicated within brief physician encounters alone. Dietitian-led care enables early identification of malnutrition and disordered eating behaviors, supports structured reintroduction strategies, and facilitates flexible dietary plans that evolve with disease activity. Contemporary guidelines increasingly recognize dietitians as essential members of the multidisciplinary IBD team, particularly for patients who modify their diet or demonstrate nutritional vulnerability [1, 9, 23]. 

Effective implementation of this paradigm requires intentional communication across four key counseling domains. First, educating patients that symptoms do not always reflect inflammation reinforces that occasional gastrointestinal discomfort does not imply disease harm [1, 2]. Second, setting expectations clarifies that diet alone rarely induces sustained remission and should complement, not replace, evidence-based medical therapy [1, 9, 23–25]. Third, reframing dietary success as nutritional adequacy, flexibility, and sustainability rather than maximal restriction [1, 12]. Finally, planning reintroduction and explicitly discussing which foods will be reintroduced, when, and how tolerance will be assessed [1, 23]. In pediatric populations, counseling should additionally prioritize growth, development, and family-centered eating patterns [13, 14, 16, 17]. 

A patient-centered paradigm must account for the substantial role that patient preferences, values, and lived experience play in dietary decision-making. Surveys consistently demonstrate that patients with IBD place high importance on diet as a modifiable factor within their control, and many report significant improvements in quality of life and psychological well-being when they feel heard and respected in dietary discussions, regardless of whether dietary changes produce objective disease benefit [3, 4, 11]. Patient-reported outcomes (PROs), including disease-specific quality of life, food-related anxiety, social functioning, and satisfaction with dietary care, represent meaningful endpoints that are often less utilized in dietary trials relative to biomarker or endoscopic outcomes. While validated quality of life instruments such as the Inflammatory Bowel Disease Questionnaire (IBDQ) have been incorporated as secondary endpoints in some dietary trials, food-specific validated PROs (such as food-related quality of life scales and ARFID screening tools) have not been systematically included in dietary intervention studies. This gap limits our understanding of how dietary strategies affect the aspects of illness experience most salient to patients. Incorporating these outcomes into research and into clinical interactions strengthens the case for individualized, collaborative dietary counseling over standardized restrictive prescriptions. Clinicians who acknowledge patient dietary concerns, explain the evidence transparently, and involve patients in goal-setting are more likely to support sustainable dietary behavior change and reduce the appeal of unsupported elimination strategies adopted outside of medical guidance.

## Gaps in Evidence and Future Research Directions

Despite growing interest in dietary therapy for IBD, substantial gaps in the evidence base remain [1, 9]. Long-term safety and feasibility data for both restrictive and inclusive diets for those with IBD are limited, and definitions of “elimination diets” vary widely across studies, complicating comparisons [9, 11, 18]. Most dietary trials rely on short-term symptom outcomes rather than objective endpoints such as mucosal healing, histologic remission, or long-term relapse prevention [9, 22]. Pediatric data are especially important given the heightened vulnerability of this population to developmental consequences [13, 14, 16, 17]. 

Future research priorities include mechanistic diet–microbiome studies, pragmatic real-world trials evaluating adherence and sustainability, pediatric-focused investigations, and equity-oriented research addressing cost, food insecurity, and access to dietitian services. Prospective incorporation of validated food-specific patient-reported outcome measures into future dietary intervention trials would substantially advance understanding of the patient experience and capture outcomes that matter most to those living with IBD [13, 18, 28–30]. Standardized dietary definitions and longer follow-up periods will be critical to advancing the field [9, 34]. 

## Conclusion

Elimination diets have become common for many patients with inflammatory bowel disease, yet their benefits remain unclear and their risks increasingly apparent. While restrictive dietary patterns may offer short-term symptom relief in select circumstances, they do not reliably induce mucosal healing or sustain remission and may contribute to malnutrition and psychosocial distress. The future of dietary care in IBD is not restriction, but in personalized, dietitian-led care that prioritizes dietary diversity and adequate nutrition. Moving beyond restriction represents not a step-back from dietary therapy, but its maturation, aligning nutritional guidance with contemporary evidence, patient-centered care, and long-term disease management.

## Key References

- Hashash JG, Elkins J, Lewis JD, Binion DG. AGA Clinical Practice Update on Diet and Nutritional Therapies in Patients With Inflammatory Bowel Disease: Expert Review. Gastroenterology. 2024;166(3):521-532. https://doi.org/10.1053/j.gastro.2023.11.303
  - ○ The most current major society guidance document from the United States on diet in IBD. Directly underpins the paper's central recommendation to shift from elimination-focused diets toward inclusion-focused diets. Endorses the Mediterranean diet as the preferred default dietary pattern for most patients with IBD.

- Svolos V, Gordon H, Lomer MCE, et al. European Crohn's and Colitis Organisation consensus on dietary management of inflammatory bowel disease. J Crohns Colitis. 2025;19(9):jjaf122. https://doi.org/10.1093/ecco-jcc/jjaf122
  - ○ The first dedicated ECCO consensus document on dietary management in IBD, representing the most comprehensive international expert synthesis to date. Discourages long-term elimination diets and reinforces the paper's recommendation for inclusive, dietitian-led dietary care.

- Ville A, McRae R, Nomchong J, et al. Effects of a Low FODMAP Diet in Inflammatory Bowel Disease and Patient Experiences: A Mixed Methods Systematic Literature Review and Meta-Analysis. J Hum Nutr Diet. 2025;38(4):e70106. https://doi.org/10.1111/jhn.70106
  - ○ The most current systematic review and meta-analysis on low-FODMAP diet in IBD, demonstrating symptom benefit without effect on disease activity or inflammatory markers.

- Gregersen L, Moos C, Hikmat Z, et al. Study Design Complexity and Participant Completion in Dietary Trials for Inflammatory Bowel Disease: A Systematic Review and Metaresearch Study. Adv Nutr. 2026;17(4):100614. https://doi.org/10.1016/j.advnut.2026.100614
  - ○ A 2026 study confirming an overall non-completion rate of approximately 16% across 62 IBD dietary trials, with markedly higher dropout in self-prepared versus meal-provided study designs. Provides the strongest available evidence that real-world adherence to elimination diets is substantially lower than trial data suggest.

## Data Availability

No datasets were generated or analysed during the current study.
